# Comment on Favresse et al. Persistence of Anti-SARS-CoV-2 Antibodies Depends on the Analytical Kit: A Report for Up to 10 Months after Infection. *Microorganisms* 2021, *9*, 556

**DOI:** 10.3390/microorganisms9081786

**Published:** 2021-08-23

**Authors:** Johannes Schulte-Pelkum

**Affiliations:** Head of Assay Design, Thermo Fisher Scientific, Phadia GmbH, 79111 Freiburg, Germany; johannes.schulte-pelkum@thermofisher.com

We thank the authors of the article [1] for their observations and recommendations, discussed in the context of the recommended cut-off for the EliA SARS-CoV-2-Sp1 IgG test (referred to by the authors as Phadia S1 IgG). The authors consider a redefinition of the manufacturer’s cut-off in order to increase the sensitivity of the test. 

The EliA SARS-CoV-2-Sp1 IgG assay is intended to identify individuals with an adaptive immune response to a recent or prior SARS-CoV-2 infection, with a focus on high specificity. High specificity for antibody tests against SARS-CoV-2 is particularly important when used in individuals who have not had a documented, PCR-confirmed SARS-CoV-2 infection. The decision on the optimal cut-off for the EliA SARS-CoV-2-Sp1 IgG test was taken in the early phase of the pandemic, with an overall infection rate of no more than 0.2%. Even today, with a global case number of 148 million [2] (resembling approx. 1.9% of the world population), the need for high specificity is evident: a diagnostic test with a high specificity of 98.1% would result in an equal amount of false and true positive test results. 

In order to develop the EliA SARS-CoV-2-Sp1 IgG assay, we tested 163 samples (serum and lithium heparin) from PCR-confirmed COVID-19 patients with the EliA SARS-CoV-2-Sp1 IgG test and set the cut-off to 10 EliA U/mL (low limit of equivocal zone set to 7 EliA U/mL). Positive percent agreement (sensitivity) was observed at 97.6% (80/82) (95% CI: 91.5–99.3%) >15 days post symptom onset (Table 1). Specificity was determined with a set of 340 serum samples collected before December 2019 from healthy blood donors. Negative percent agreement (specificity) was observed at 99.4% (338/340) (95% CI: 97.9–99.8%) using the low limit of the equivocal zone, as shown in Table 2 [3].

We recognize that in cases where an individual has a documented PCR-confirmed SARS-CoV-2 infection, high specificity may be less of a concern. To address questions depending on high sensitivity rather than on high specificity, including longitudinal studies of patients with a known infection history, the cut-off can be set to 97.9% specificity at 0.7 EliA U/mL, the detection limit of the test. This modification resulted in a sensitivity of >99% in an internal study with 694 longitudinal samples 2–27 weeks post symptom onset (Table 3 and Table 4). The control group consisted of 478 samples including 330 healthy blood donors, reflecting all ethnicities in the US, and 148 infectious disease samples.

An interesting observation in the long-term monitoring of anti-SARS-CoV-2 immunity was that, despite a significant drop in anti-Spike 1 IgG titers over a period of up to 6 months, strong virus-neutralizing activity was still measurable with an ACE2 receptor binding inhibition assay. These findings may indicate the importance of including assays to measure the neutralizing potential of anti-SARS-CoV-2 antibodies in long-term follow-up studies. This approach could help to answer the perennial question of immunity to reinfection and its longevity.

## Figures and Tables

**Table 1:** Positive Percent Agreement (PPA).

Days Post Onset of Symptoms	Number of Samples Tested	EliA SARS-CoV-2-Sp1 IgG Positive	EliA SARS CoV-2-Sp1 IgG Negative/Equivocal	Positive Percent Agreement [%]	95% CI [%] Wilson-Score Method
0–7 days	26	4	19/3	15.4	6.2–33.5
8–14 days	55	32	20/3	58.2	45.0–70.3
≥15 days	82	80	2/0	97.6	91.5–99.3

**Table 2:** Negative Percent Agreement (NPA).

Group	Number of Samples Tested	EliA SARS-CoV-2-Sp1 IgG Positive/Equivocal	EliA SARS-CoV-2-Sp1 IgG Negative	Negative Percent Agreement (95% CI) [%] Wilson-Score Method
Blood donors	330	1/1	328	
Pregnant women	10	0/0	10	
Total	340	1/1	338	99.4 (97.9–99.8)

**Table 3:** Stratification of samples from 694 COVID-19 patients, and 478 healthy Controls at 0.7 EliA U/mL cut-off and 97.9% specificity in an internal study.

Class	EliA SARS-CoV-2-Sp1 IgG		Total
	>0.7 EliA U/mL	≤0.7 EliA U/mL	
COVID-19	690	4	694
Controls	10	468	478

**Table 4:** Sensitivity and specificity of EliA SARS-CoV-2-Sp1 IgG (Phadia S1 IgG) at 0.7 EliA U/mL cut-off and 97.9% specificity in an internal study.

	Proportion	Wilson 95% CI
PPA (Sensitivity)	99.4%	0.985–0.998
NPA (Specificity)	97.9%	0.962–0.989
